# Agroextractivism in Argentina environmental health, scientific agendas, and socioecological crisis

**DOI:** 10.3389/fpubh.2023.1304514

**Published:** 2023-12-01

**Authors:** Cecilia Gárgano

**Affiliations:** Laboratorio de Investigación en Ciencias Humanas, Universidad Nacional de San Martín, Consejo Nacional de Investigaciones Científicas y Técnicas, Buenos Aires, Argentina

**Keywords:** agriculture, agroextractivism, environmental health, scientific knowledge, socioecological crisis, agroextractivism, environmental health

## Introduction

Today agribusiness occupies 70–80% of the global arable land [(1), p. 55]. Several authors have conceptualized this agroindustrial production as agroextractivist that has consolidated a regime of specialization in monocultural export commodities (palm oil, soybean, sugarcane, avocado, among others) and biofuel generation (2–4).

This production consumes a big chunk of the world's oil reserves while generating between 20 and 30% of greenhouse gases influencing global warming [(5), cited in (6), p. 460]. What has been called the twenty first-century agro-extractivism [(7), p. 7] is the enhanced use of fossil fuels, pesticides and chemical fertilizers and the over-consumption of fresh water to expand the agricultural frontier (displacing other crops, native forests, and populations) while boosting massive wealth concentration.

According to Giraldo (8), there are many examples of agroextractivism but the case of soy is paradigmatic. To produce one ton of soybeans, kilograms of minerals (magnesium, sulfur, phosphorus, potassium, nitrogen) must be extracted that are not replenished in the soil and thus it degrades, rapidly undermining the reproduction of life [(8), p. 16].

In 1996, during the second presidency of Carlos Menem, Argentinean agriculture's neo-liberalization began with the authorization of the use of a new transgenic soybean variety: RR soybean (RoundUp Ready, RoundUp Resistant), a variety modified through transgenesis.

Currently, the United States, Brazil, Argentina, and Canada account for 83% of the world's genetically modified crops, followed by India, China, Paraguay, South Africa, Uruguay, and Bolivia. Around 24 million hectares, 12–13% of the world area cultivated with transgenics, corresponds to practically all soybean, cotton, and 98% corn production (9).

I will argue that agro-extractivism in Argentina is a major contributor to the socioecological crisis and a threat to public health, and that it is necessary to promote agroecology as an alternative model. In the absence of official statistics on the quantities of pesticides used in Argentina, we maintain that it is necessary to promote research that documents the environmental and health impacts of this agriculture. Furthermore, expanding state capacities for research and development in the fields of environmental health and agroecology can be a strategy to promote the transformation of current production and consumption patterns.

## Discussion

### Environmental health, scientific agendas, and agroextractivism

The most widespread genetically modified crops in the world are Bt cotton and Bt maize, modified to resist pests by introducing genes from the soil bacterium Bacillus Thuringiensis, which provides resistance to insects, along with Roundup Ready soybeans, modified to survive applications of glyphosate-based herbicides, originally Roundup (10). Both strategies, the introduction of insecticidal genes and the development of herbicide-tolerant varieties, have led to processes of biological resistance in pests and weeds they seek to combat (11).

The technology package includes the agronomic management of “no-till”, the genetically modified crops and the agricultural chemical inputs (mostly herbicides) to which they are tolerant. Seeds and technology are patented and commercialized chiefly by a few multinational firms creating problems of knowledge concentration and inequality (12).

Contamination of water sources, soil, and air by pesticides, as well as soil degradation due to the lack of crop rotation, have been the main environmental implications associated with these agricultural practices (13). Specialization in large-scale monocultures has already reduced the genetic diversity present in agricultural systems [(14), p. 75]. In this scheme, transgenic crops (mainly soybean and corn, representing around 180 million hectares cultivated worldwide) and biofuels play an essential role [(1), p. 57].

As for health-related damages, international literature has correlated pesticide exposure with the occurrence of spontaneous abortions, birth defects and genetic damage (15); Hodgkin's lymphoma and leukemia (16–18), Parkinson's disease (19); endocrine disorders (20, 21), semen quality impairments (22), respiratory conditions, autism (23), various types of cancer (24–26), and respiratory conditions associated to pesticide exposure (27–31). The health effects derived from the intensive use of pesticides are already a public health alert [(32–36), among many other available studies].

The effects of intensive pesticide use on various health issues in Argentina have been raised as concerns by healthcare professionals, researchers, and affected communities [(37–41), among others]. The primary warnings and demands have come directly from the affected communities themselves.

The Italian Hospital of Buenos Aires was founded in 1853. It is a private, high-complexity university hospital. Following the parameters of the One Health approach, the Hospital formalized the Environmental Health consulting office and the measurement of glyphosate levels, giving answers to the demand of patients who associate their signs and symptoms to environmental pollution.

The concept of One Health has emerged, recognizing the systemic interdependence and the changes in human health that are expressed synchronously and indivisible from the environment (42, 43). According to the ecosystem approach recently articulated by the WHO under “One Health,” the sustainability of human health is inseparable from the health of animals, plants, and microorganisms, as well as the sustainability of all complex subsystems that make up our environment, primarily those related to water and the oxygen cycle (44).

In 2013, the Italian Hospital's Research Program in Health and Environment was formalized, and it began various lines of research in environmental health. For 10 years, they recorded the increase in patient consultations from rural areas. The first participatory action research was funded by the National Cancer Institute and included the validation of an analytical methodology for quantifying glyphosate in urine samples (using liquid chromatography coupled with tandem mass spectrometry) (45). Other analytical developments were made possible, such as the measurement of chlorpyrifos in umbilical cord blood and bisphenol A in urine and blood.

Finally, in June 2022, the Italian Hospital formalized the Environmental Health clinic. One of its objectives is to innovate in the evaluation of epigenetic changes associated to environmental contaminants exposure, and investigate prenatal and perinatal exposure to environmental factors in the development of diseases in adulthood, for which there is limited longitudinal data (42).

The Italian Hospital is a private institution and that it is taking care of a public health issue that should also be addressed by the Argentinean State. However, the Argentine State has not compiled official statistics or conducted epidemiological surveys on the health impact of pesticides on the population. There are also no official systematic environmental studies in place. This situation is directly linked to the absence of official data on the quantities of pesticides used and the presence of regulatory gaps. There are no regulations that establish threshold values of the most commonly used pesticides to establish the safety of drinking water. Additionally, there are no national laws that specify the distances from watercourses, homes, and rural schools at which agrochemical applications should be conducted (46, 47).

On the other hand, state scientific research agendas have been promoted to generate new crop varieties tolerant to herbicides. In October 2020, the Argentine government approved the first domestically produced transgenic wheat: the HB4 variety. It was modified to be drought resistant, so it was presented as a national scientific contribution to the climate crisis and a commitment to sustainability [(48), p. 45]. The pillars favoring this liberalization were: state financing, the participation of national capitals and the potential foreign currency income.

To obtain the Hb4 wheat, drought resistance was obtained by transferring the HaHb4 gene naturally present in sunflower, generating that the plant does not register water stress and continues to grow. In addition to this characteristic, the crop was modified to be tolerant to the herbicide Glufosinate Ammonium, whose toxicity is superior to that of Glyphosate [(49), p. 11]. This herbicide is produced by Bioceres, the same company involved in the technological development of the new wheat.

This case can be understood in the context of the hegemonic scientific model, as Rikap et al. [(50), p. 2] analyzes, the dominant conceptions of scientific production and its material conditions contribute to deepening the humanitarian crisis. It is important to stress that Argentina is today placed high in the world's ranking of pesticide use, which has severe consequences on water, soil, air and human bodies (51).

The official discourse is that this wheat variety will contribute to reducing the use of herbicides through better soil management thanks to the soybean/wheat alternation, which would result in more sustainable agriculture. However, recent history indicates the opposite.

Despite the promise of a reduction in chemical inputs that accompanied the arrival of these crops, between 1990 and 2012, the use of herbicides increased by 12.79% in Argentina [(52), p. 3]. From 1996, when the first transgenic crop was approved, to 2020, 62 transgenic crops were authorized in the country; 80.64% were designed to be pesticide tolerant (53).

Despite the existence of a large number of historical examples throughout the world, as well as literature that for years has shown that this type of agriculture poses a risk to food security (54, 55), a large number of discourses, agricultural practices and techno-scientific research persist in deepening its productive dynamics. New technological solutions are offered as salvation in the face of an imminent gap between population and resources, updating old Malthusian ideas [(56), p. 457] while introducing new strategies based on sustainability discourse.

However, this is not the only type of agriculture that exists in the country. In Argentina, there are 4.800 organic and agroecological farming establishments covering over four million hectares. With diverse production, ranging from horticulture to grains and from honey to livestock, the sector holds enormous potential, driven by peasant families and small-scale farmers (57).

Conversely, multiple studies have systematized how agroecological practices and peasant agriculture knowledge can generate successful tools for developing climate resilience and territorial health. In this line, Nicholls and Altieri (1) argue that traditional agricultural systems offer a wide range of practices that increase functional biodiversity in crop fields and thus contribute to the resilience of agroecosystems, such as crop diversification (polycultures), preservation of local genetic diversity, animal integration, organic matter employment, water harvesting, and agroforestry systems.

According to international organizations such as the FAO (Food and Agriculture Organization), non-intensive family farming is responsible for a large part of food production worldwide (58). At the same time, this agriculture offers solutions to problems derived from global warming. In 57 nations, agroecological projects covering 37 million hectares (equivalent to 3% of the total cultivated area in these countries) were shown to increase average crop yield by 79%, as well as land productivity on 12.6 million farms [(59), p. 1115].

More than 10 Argentine provinces and different departments of Uruguay already have municipalities in which peasant agriculture has expanded. Beyond establishing another relationship between farms and the land—influencing collective and environmental health—this type of agriculture has reduced the high costs imposed by the technological package based on intensive pesticides [(60), p. 51].

On the other hand, agribusiness is associated with the concentration of land access and usage. In Argentina, this has been linked to the reduction of traditional activities of the peasant economy and/or small producers, such as goat and sheep farming and horticulture [(61), p. 425]. Comparing the data collected by the 2002 and 2018 National Agricultural Censuses, it is observed that 25% fewer EAPs (“explotaciones agropecuarias”, the agricultural holding) were registered in less than two decades. An investigation showed that the total number of EAPs registered in the 2018 CNA was 250,881 units, compared to 333,533 in 2002, which implies the disappearance of 82,652, approximately a quarter, at an average annual elimination rate of 5,166 EAPs [(62), p. 14].

Agroextractivism is a major contributor to the socioecological crisis and a threat to public health. On the contrary, agroecological proposals have the capacity to favor climate resilience, promote socioecological diversity and generate healthy food that does not depend on external chemical inputs (63–65).

Promoting a public agenda that takes into account environmental health from a comprehensive perspective is crucial in this scenario. Likewise, expanding state research and extension agendas to support agroecological experiences appears to be a necessary challenge.

In summary, we believe that it is necessary to strengthen state scientific and technological capabilities focused on agroecology and environmental health as a strategy that can contribute to transforming current production and consumption patterns.

## Author's note

A preliminary version of this document was published as the policy brief “Argentina en el contexto de crisis socioambiental global ¿Más agro-extractivismo para salir de la crisis extractivista?” as a result of my research stay at the University of Kassel within the Extractivism Project.

## Author contributions

CG: Conceptualization, Investigation, Methodology, Project administration, Writing – original draft, Writing – review and editing.
